# Reconnecting Anisometropic Amblyopic Eyes to the Cortex: VEP-Based Auditory Biofeedback

**DOI:** 10.3390/diagnostics14171861

**Published:** 2024-08-26

**Authors:** Iulia-Andrada Nemeș-Drăgan, Alexandru Țîpcu, Mădălina Claudia Hapca, Raluca Pașcalău, Simona-Delia Nicoară

**Affiliations:** 1 Department of Ophthalmology, “Iuliu Hatieganu” University of Medicine and Pharmacy, 400012 Cluj-Napoca, Romania; madalina.prodan06@gmail.com (M.C.H.); simonanicoara1@gmail.com (S.-D.N.); 2 Ophthalmology Clinic, Emergency County Hospital, 3-5 Clinicilor Str., 400006 Cluj-Napoca, Romania; raluca.pascalau@yahoo.ro; 3 Department of Radiotherapy, “Ion Chiricuță” Institute of Oncology, 34-36 Republicii Str., 400015 Cluj-Napoca, Romania; alextipcu@gmail.com; 4 Doctoral School of Medicine, “Iuliu Hatieganu” University of Medicine and Pharmacy, 8, V.Babes Str., 400012 Cluj-Napoca, Romania

**Keywords:** amblyopia, anisometropia, visual rehabilitation

## Abstract

Objective: This study aimed to evaluate the effectiveness of a visual rehabilitation method for anisometropic amblyopia that uses visual evoked potential (VEP) parameters and sound biofeedback to increase objectiveness. Design: an observational, case-controlled trial. Setting: Ophthalmology Clinic, Emergency County Hospital, Cluj-Napoca, Romania. Participants: Sixteen subjects with amblyopic anisometropia, aged 15–57, and sixteen controls, aged 24–33, were included. Interventions: Subjects were divided into two groups. The control group, composed of normal-vision subjects, and the amblyopic group received 10 training sessions. The rehabilitation program lasted 10 min, took place twice a week, and ran over five consecutive weeks. During each session, the subjects were asked to fixate on a target on the computer screen and were instructed to keep the fixation and maintain the sound of the biofeedback at high frequency. Main Measures: The study assessed the main visual parameters at baseline, after 10 sessions, and 1, 3, 6, and 12 months after treatment. Performance was evaluated by measuring visual acuity, contrast sensitivity, and reading velocity (words/minute). Results: In the experimental group, mean BCVA improved with two rows, which means an improvement in the LogMARLogMAR scale with an average of nine letters. These values tended to be maintained over time. Both groups showed better reading velocities after training, but this parameter has undergone large variability during follow-ups. Contrast sensitivity was also improved and stable. Conclusions: Visual rehabilitation with the Retimax Vision Trainer can improve visual performance in patients with amblyopia after the critical period, an improvement that is maintained in most cases for at least one year after treatment.

## 1. Introduction

Amblyopia can be defined as unilateral or bilateral perceptual and neuro-visual impairment due to any cause that creates a disparity of vision between the two eyes [1]. Worldwide, up to 90% of patients with amblyopia have strabismus and/or anisometropia [2]. For a long time, it was considered to be mostly a cortical disturbance due to abnormal input during the critical period since no organic causes could be found upon physical examination of the eye [3]. Currently, microstructural findings advocate for abnormalities of the optic nerve and suggest that amblyopia is not a purely cortical disorder [4]. Electrophysiological studies performed in children outside the critical developmental period showed significant improvement in retinal ganglion cell (RGC) activity and an increase in wave amplitudes after an amblyopic treatment [5]. The exact pattern of perceptual deficits seems to differ depending on the etiology, thus the definite impact on visual function improvement is determined by changes at multiple locations on the visual pathway [6].

Adults’ visual system plasticity has been studied for many years together with many perceptual learning techniques. The newly developed training approaches try to use methods of learning that can increase visual performance together with objective tests. Nowadays, there is evidence that demonstrates that any attempt at treatment of amblyopia after the critical period is worthwhile [7,8,9,10,11]. Most conventional therapies used for old amblyopic patients do not offer objective feedback. The voluntary control of a physiological activity together with information about it is what defines biofeedback [12]. An important factor for the success of a therapy that may be quantified and improved is visual attention. The ability to maintain focus and to keep visual attention on a certain target can be demonstrated by performing steady-state visual evoked potentials (SS-VEPs) [7,13,14]. The electrophysiological parameter that improved after ophthalmological or psychological perceptual learning techniques was the amplitude of the waves.

The aim of this study was to evaluate the improvements in visual performance that can appear after completing 10 training sessions performed with a device equipped with a real-time feedback system in amblyopic patients after the critical period and the persistence of the results.

## 2. Methodology

The study design was an observational, case-controlled trial. This study was approved by the Medical Research Ethics of the “Iuliu Hatieganu “University of Medicine and Pharmacy “- Cluj-Napoca, Romania (file number:102/31.05.2024) and the Institutional Review Board of the Emergency County Clinical Hospital from Cluj-Napoca. All participants provided their acceptance by signing an informed consent form to confirm their participation in the research.

## 3. Sample

A total of 16 patients with high monocular anisometropic or mixed (strabismic and anisometropic) amblyopia were enrolled in the study. Their ages ranged between 15 and 57 years. A total of 16 controls aged between 24 and 33 years without any ocular pathology were also included in the visual training sessions. All the participants were thoroughly examined by an ophthalmologist before the start of the first session. The examination included best corrected visual acuity (BCVA), cycloplegic refraction (Tropicamide 1% 1 eye drop three times), orthoptic assessment, slit lamp examination, and fundoscopy. Diagnostic tests including optical coherence tomography or electrophysiology were performed to exclude other ocular pathologies that could influence the results of our treatment or other causes of low vision.

## 4. Training Protocol

**Figure 1:**
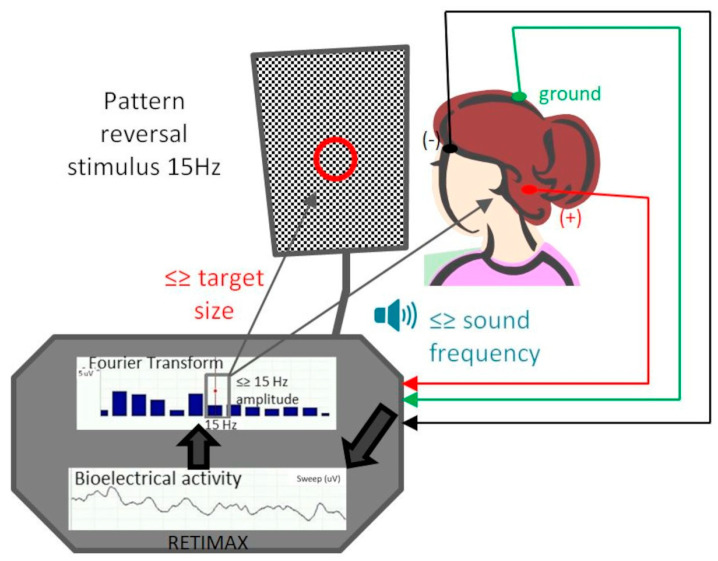
Schematic illustration of the training protocol. The vision trainer records visual evoked potentials based on monocular stimulation with a pattern reversal stimulus of 15 Hz frequency. Based on signal amplitude, it provides auditory feedback as a sound of which frequency varies proportionally with this amplitude and visual feedback by decreasing the target size with increasing amplitude. The patient is encouraged to make the sound higher and the target smaller. The VEP active electrode is placed in the position of the visual cortex (red wire, +), the negative electrode (black wire, −) is placed on the forehead, and the green wire is the ground electrode.

Visual rehabilitation sessions were performed using the Retimax Vision Trainer (CSO, Florence, Italy), a noninvasive rehabilitation method for improving visual performance in amblyopic patients by means of the detection of visual evoked potential (VEP) and auditory and visual biofeedback. Its main use is for electrophysiological studies, but it also enables us to obtain VEP examinations and real-time analysis during vision training. The protocol included 10 sessions, 2 per week, for a total of 10 min per session. Participants were seated in a dark, acoustically isolated room in front of a 42-inch monitor that had a central fixation target surrounded by black and white squares with pattern reversal, at a viewing distance of 1 m. The good eye was patched, and the patient wore the proper optical correction. Skin electrodes (Ag/AgCl) were applied as they are in VEP recording, namely, active electrode Oz on the occipital scalp over the visual cortex, the reference electrode at 2/10 between nasion and inion, and ground electrode Cz on the forehead. The fixation target was perceived by each patient prior to every session. By seeing the squared pattern, a bioelectrical signal is produced in the retina, visual pathways, and cortical areas. Detection of the signal is performed with electrodes and processed by the device with a Fast Fourier Transform. The amplitude at 15 Hz on the Fourier graphwhich is the pattern reversal frequency, characterizes the cortical activity triggered by the stimulus. The device returns an acoustic feedback of a frequency directly correlated to this amplitude. The size of the fixation target decreases in reverse correlation to the amplitude, thus also providing visual feedback (Figure 1).

**Figure 2:**
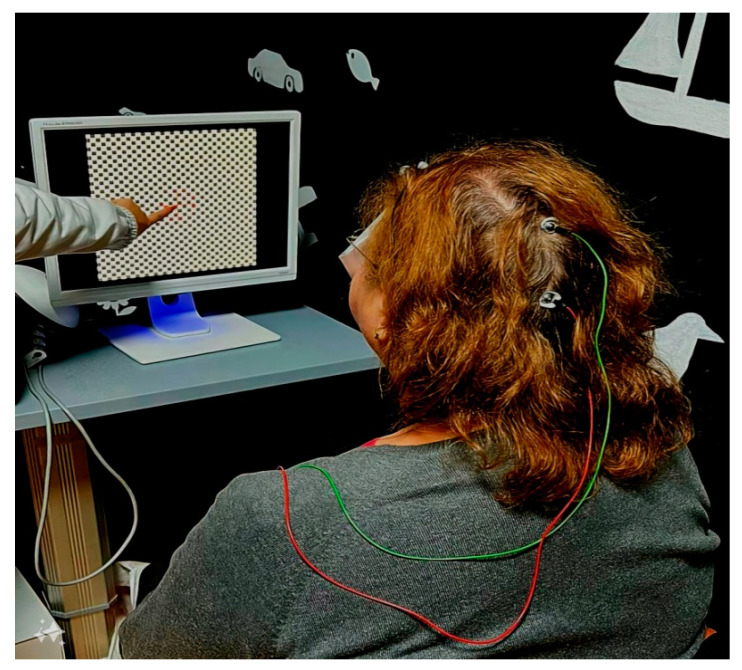
During the examination, the patient looked at the monitor with the dominant eye covered and was encouraged to maintain fixation every time the sound became lower.

Fixing the central target was of utmost importance; therefore, during the session, the operator had the role of encouraging the patient to find the fixation target and maintain it (Figure 2). Every time the patients fixated with the fovea, the bioelectrical signal detected the increase in amplitude and the background sound got higher and the fixation target got smaller. The results of each session were recorded and stored.

## 5. Visual Parameters Assessment

We evaluated the visual function at baseline, at the end of the 10 sessions, and 1, 3, 6, and 12 months after treatment in amblyopic patients and after 10 sessions in the control group. For the assessment of visual acuity, we used the LogMAR chart. A near Pelli Robson contrast sensitivity chart was used. The reading speed was measured using a printed text with Arial 14 characters. During the measurements, we asked the patient to wear their corrective spectacles to obtain the best vision correction.

## 6. Statistical Analysis

We reported our results via the mean ± 2SD (standard deviation) and median. When comparing metrics from different time points, we used Wilcoxon Signed Rank tests for pairwise comparisons and Friedman’s test for overall shift analysis. Group comparisons were performed using Mann–Whitney U testing. Linear relationships were assessed using Spearman correlations, followed by linear regressions. Adjustments for multiplicity were conducted using Bonferroni correction where applicable. P-values lower than 0.05 were considered statistically significant (alpha = 0.05). All statistical analyses were performed using IBM SPSS Statistics v.26.0.0.

## 7. Results

Based on our inclusion criteria (see the Methodology above), we selected two groups of patients:

Sixteen eyes of healthy subjects with a mean age of 28.5 ± 7.4, ranging from 24 to 33 years, and BCVA of −0.1 to −0.2 LogMAR were included in the control group (without amblyopia). The mean interocular refractive difference calculated as the spherical equivalent was lower than 0.25 D.

Sixteen eyes of amblyopic patients with a mean age of 32.9 ± 3.2, ranging from 15 to 57 years, and BCVA of 1.4 to 0.4 LogMAR were included in the amblyopic group. The mean interocular refractive difference calculated as a spherical equivalent was 3.5D; this group consisted of 12 (75%) subjects with high amblyopia and 4 (25%) subjects with medium type. Regarding amblyopia etiology, 13 (81.25%) patients were pure anisometropic and 3 (18.75%) were diagnosed with mixed amblyopia (strabismus and anisometropia). All amblyopic subjects were corrected with glasses. The prescription was recommended according to cycloplegic refraction.

### 7.1. BCVA

Individual BCVA was measured at baseline, post-training, 1 week post-training, 1 month post-training, 3 months post-training, 6 months post-training, and one year post-training for amblyopic eyes and at baseline and post-training for controls. Mean BCVA improved with two rows, which means an improvement in the LogMAR scale with an average of nine letters. Our analysis showed that age does not influence the visual outcome. The follow-up values tended to be maintained over time for the amblyopia group (Table 1)

**Figure 3:**
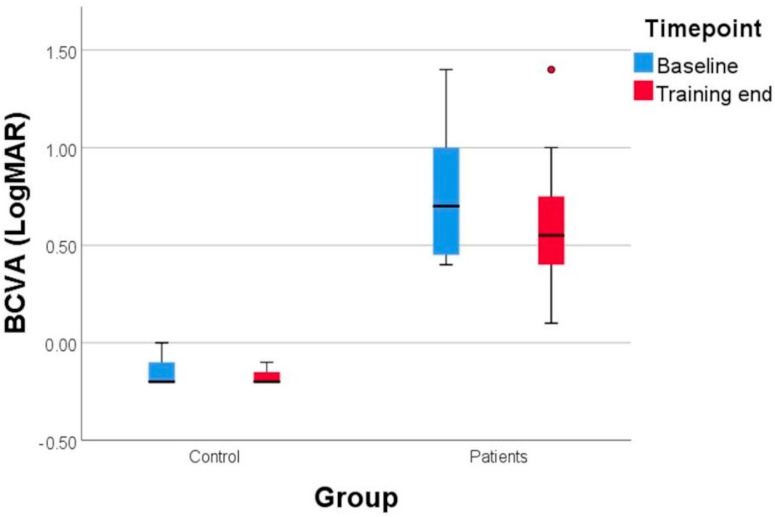
The evolution of BCVA (best corrected visual acuity) in amblyopic eyes and control eyes. At the end of the training, the amblyopic subjects showed an improvement of 9 letters, measured on LogMAR scale. The control group also presented a mild gain from −0.1 to −0.2 LogMAR.

The control group showed no significant improvement regarding BCVA. There were some situations in which the control subjects were −0.1 LogMAR and improved post-training to −0.2 LogMAR (Figure 3).

### 7.2. Reading Speed (RS)

The mean reading velocity was 174.5 ± 8.3 words/min in controls. After 10 training sessions (TS), the reading speed improved to a mean of 221 ± 8.9. In the experimental group, the baseline RS was 72.4 ± 15.5 with a subtle improvement of 77.13 ± 16.3 after rehabilitation sessions. This parameter underwent undergone large variations during follow-ups (Table 1).

### 7.3. Contrast Sensitivity (CS)

Both groups improved this parameter after 10 training sessions. Experimental subjects had a baseline mean of 1.7 and improved up to 1.9. This improvement was stable over time (Table 1). All the controls had a very good CS, with a mean value of 2 at baseline.

### 7.4. SS-VEP

**Figure 4:**
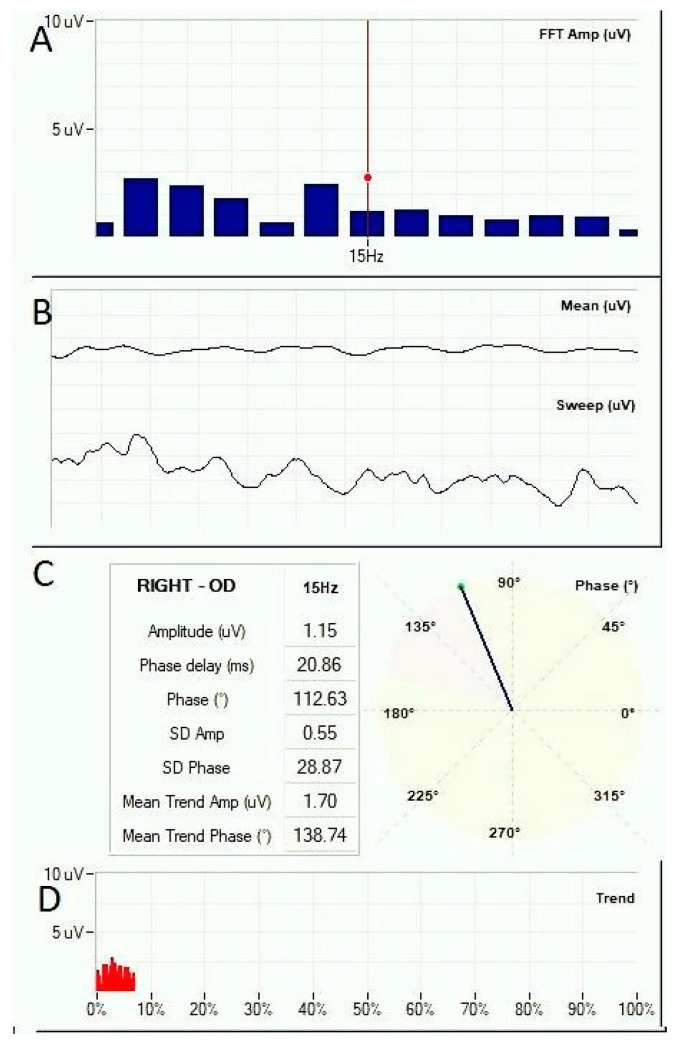
Snapshot of the Vision Trainer software 7.1.0 (Retimax, CSO, Firenze, Italy) during a training session. The SS-VEP waves are represented in the middle panel (**B**) in live signal form (Sweep) and averaged signal for the entire recording up to the live time point (Mean). Amplitude is represented on y-axis (uV) over time on x-axis (ms). Panel (**A**) shows the Fast Fourier Transform at the live time point represented in amplitude on y-axis (uV) over frequency on x-axis (Hz). Panel (**C**) presents VEP characteristics at 15 Hz, corresponding to the activity triggered by the 15 Hz pattern reversal stimulus. The first three values (Amplitude, Phase delay, and Phase) refer to the live time point, while the last four are standard deviation (SD) and mean values for the entire recording up to the live time point. Phase represents the cortical activity sinusoid shift from the stimulus wave. It is represented in degrees (also graphically on the left) as well as in time (ms). Panel (**D**) represents the amplitude trend across the training session, represented on x-axis as completion percent.

We analyzed the amplitudes of the SS-VEP (Figure 4) during the sessions and compared them with the Friedman test, which demonstrated an overall variation for the amblyopic group.

For the experimental group, there was a significant decline in the fourth session with a recovery that was stable until the last one. As expected, there was a tendency for lower amplitudes in subjects with a high amblyopia, but without being significant, likely due to the low number of patients (Table 2). The age of the subject seemed to influence the SS-VEP amplitudes (Table 2). Individuals aged above 35 presented higher amplitudes without significance because of the reduced number of subjects. There was a strong positive correlation between the anisometropic grade and the BCVA increase measured with LogMAR with a Spearman coefficient of 0.693 (*p* = 0.003). A moderate-strong correlation was found with the same test between the high amplitudes in SS-VEP and an improvement in BCVA. For every microvolt gained during the sessions, LogMAR VA improved by 17.34%. In absolute values, a decrease of 0.14 represents seven letters on the LogMAR chart.

## 8. Discussion

Our report is the third to investigate the effects of the Retimax Vision Trainer on visual function in amblyopia. Other studies did not report any follow-up at certain time points after perceptual learning training cessation. In this study, both the control and experimental groups exhibited improvements in all measured variables, including visual acuity, reading speed, and contrast sensitivity after 10 sessions of training. The results obtained in the amblyopic patients lasted for 1 month, 3 months, 6 months, and even one year in the follow-up. The improvements were accompanied by objective features in terms of the SS-VEP amplitude after 10 training sessions. As far as we know, this is the first study using objective feedback (SS-VEP) that had a control group. Even though there is a large debate in the literature regarding the critical period, we consider it after the age of 8 years old, thus we started training after this period and only in patients who had previous occlusion [15,16]. The mean BCVA improvement of 0.14 LogMAR (7 letters) among the subject group is more consistent than other similar studies that used the same device for Visual Training [5,7]. Most of the studies that use a perceptual learning method for amblyopia treatment showed an improvement in LogMAR VA that ranged between 0.08 and 0.24 [17]. The Retimax Visual Trainer (RVT) seems to also quantitatively improve the visual performance in subjects with good VA and without amblyopia. Tranchita et al. used RVT for neuro-visual enhancement for athletes with normal VA who wanted to improve their visual and sports performance [18].

The age of the subjects did not influence the visual acuity improvement, even though the amplitudes of SS-VEP were directly proportional to age. There is well-known evidence of plasticity in visual attention and learning in the adult visual cortex, which was demonstrated in normal subjects [19,20].

Having different pathogenetic mechanisms, the two types of amblyopia also have different manifestations; namely, strabismic amblyopia is characterized by impaired contrast sensitivity and high positional uncertainty, while anisometropic amblyopia consists of reduced spatial resolution [21]. Due to our small sample, we could not demonstrate a worse outcome in mixed amblyopia than in anisometropic type. Nevertheless, Woodruff, G. et al. showed that pure anisometropes had better outcomes than pure strabismic and mixed amblyopia [22,23]. In the treatment of unilateral amblyopia using penalization methods, the type of amblyopia does not seem to have a significant influence on the visual outcome, although eccentric fixation was correlated with poor results [24,25]. Similarly to other studies, considering the training duration and the subjects’ availability, we considered that 10 sessions were enough to learn. There were 2 subjects for whom we extended the protocol to 20 sessions, but none of the measured parameters improved. Hussain et al. showed that perceptual learning experiments can be influenced by training duration in adults [26]. In contrast, other authors concluded that the training duration was not significant in visual acuity improvement regardless of treatment methods [27]. Pre-treatment visual acuity was not a significant factor in the success of the therapy in comparison with occlusion, where the depth of amblyopia is a predictive factor of the outcome [23,28]. Visual attention was an important factor that seemed to influence the amplitudes in SS-VEP in our subjects. Patients aged above 36 had higher amplitudes. There are several studies that confirm the deficits in visual attention in amblyopic adults and children during monocular and binocular vision [29,30,31]. Animal and human electrophysiology studies support the view that attentional processes might be involved in amblyopic deficits [32]. Besides its fundamental role in amblyopia treatment, it was proven that attention modulates long-term plasticity in the early visual cortex in visually normal subjects [33]. To objectivize the visual attention aspect of the training, we recorded SS-VEPs as a well-established tool for studying cognitive mechanisms. As an added value to other previously established vision training methods, we emphasize the importance of staying focused. It was reported that amblyopic eyes have smaller pattern-reversal VEP amplitudes with a significantly higher latency, proportional to visual acuity [34]. There is a normal interocular amplitude VEP difference and latency between normal and anisometropic subjects, while those with associated strabismus show higher implicit time difference between eyes, suggesting that the neural deficit in mixed or strabismic amblyopia could be more severe than in anisometropic type [35,36]. In accordance with these authors, we found a correlation between SS-VEP amplitudes and the increase in visual acuity after 10 training sessions. Namely, for every extra millivolt, LogMAR improved by 17.34%. In absolute values, this represents 0.14 or 7 letters. There are also authors who did not find any difference between VEP values in anisometropic compared to strabismic ambylopes [37]. Wang Y et al. found that in anisometropic amblyopia, the P100 wave amplitude is influenced by the checkerboard stimulus size, while in strabismic amblyopia, it is not [21].

Similar to the results published by Esposito et al. and Lapajne et al., all visual parameters improved after 10 training sessions in controls and amblyopic subjects with lasting effects on visual acuity, but with a relapsing effect regarding the reading speed parameter. Reading performance is a broader clinical indicator of visual capacity that can be impaired in amblyopia due to discordant information between the eyes [38,39,40]. Some limitations and strengths of the study must be taken into consideration. First, the sample is small—although it is larger than others that have used Retimax [5,7,41]. In comparison with other similar studies, we used a control group, but without long-term follow-up. The subjects involved in the study were evaluated and performed the training during the morning when the ability to sustain a high level of attention is maximum [38,42]. Improvements in visual tasks after perceptual learning have been demonstrated with electrophysiological examinations, and significant improvement of the P50 wave on pattern electroretinography before and after treatment was recorded in amblyopic subjects [5]. Previous studies have shown that it can also be evaluated using VEP or structural and functional Magnetic Resonance Imaging (MRI/fMRI) [38]. Wang X et al. described a direct correlation between primary visual cortex activation documented by functional MRI and P100 wave amplitudes on VEPs in patients with amblyopia [21]. Occlusion therapy was also shown to cause increased cortical activation on fMRI [43]. The two types of amblyopia, strabismic and anisometropic, were shown to have different cortical activation deficit patterns on fMRI [38]. There are also important differences in fMRI activation profiles between adults and children with amblyopia. As a result, it is expected that perceptual learning may involve different neuronal circuits in these categories of patients, partially explaining their different responses. In our study, amblyopia was diagnosed and perceptual learning outcomes were measured based on clinical findings. Differential diagnosis between strabismic and anisometropic amblyopia was made based on patient history and refraction. Functional documentation using PERGs, VEPs, and fMRI, as well as the assessment of brain connectivity with tractography, could have added more nuance and mechanistic explanations to our clinical outcomes.

In conclusion, according to the results of this study, the tested technique can improve visual performance in patients with amblyopia after the critical period, an improvement that is maintained in most cases for at least one year after treatment. This proposed protocol also had a beneficial effect on healthy subjects by improving the reading speed. Unlike other perceptual learning methods, Retimax uses real-time SS-VEP, an objective way to assess subjects’ attention during training sessions and confirm good understanding.

## Figures and Tables

**Table 1:** Comparison of all measured variables of the amblyopic eyes before and after treatment. BCVA = best corrected visual acuity. Friedman’s = Friedman’s test for overall shift analysis. WSR = Wilcoxon Signed Rank tests for pairwise comparisons.

Measure	Pre-Treatment	After 10 Sessions	1 Year Follow-Up	*p*-Value (Friedman’s)	*p*-Value (WSR)
BCVA (LogMAR)	0.775 ± 0.717 (0.7)	0.588 ± 0.656 (0.55)	0.581 ± 0.756 (0.45)	<0.001 ^a^	Pre vs 10s: 0.009 ^a^
					Pre vs. 1y: 0.003 ^a^
Contrast sensitivity	1.718 ± 0.75 (2)	1.921 ± 0.396 (2)	1.921 ± 0.436 (2)	0.008 ^a^	Pre vs. 10s: 0.016 ^a^
					Pre vs. 1y: 0.055 ^b^
Reading speed (words/min)	72.44 ± 124.278 (53.5)	77.13 ± 130.7 (63)	71.88 ± 127.86 (55.5)	0.064 ^b^	Pre vs. 10s: 0.432 ^b^
					Pre vs. 1y: 0.726 ^b^

^a^ Significant difference at the 0.05 level. ^b^ Non-significant difference.

**Table 2:** Factors that can influence visual evoked potential amplitude. Linear relationships were assessed by Spearman correlations, followed by linear regressions. SS-VEP = steady-state visual evoked potentials.

Variable		Mean SS-VEP Amplitude (μV)	*p*-Value
Age (years)	Under 35	1.336 ± 1.082	0.125 ^a^
	Over 35	1.981 ± 2.104	
Amblyopia grade	Medium	1.804 ± 1.348	0.379 ^a^
	High	1.556 ± 1.816	
Amblyopia cause	Anisometropia	1.581 ± 1.808	0.459 ^a^
	Strabismus	1.779 ± 1.210	

^a^ Non-significant difference.

## Data Availability

The original contributions presented in the study are included in the article, further inquiries can be directed to the corresponding author.
